# HPV related p16^INK4A^ and HSV in benign and potentially malignant oral mucosa pathologies

**DOI:** 10.1186/s12903-024-04105-z

**Published:** 2024-03-18

**Authors:** Irena Duś-Ilnicka, Agnieszka Hałoń, Andrea Perra, Małgorzata Radwan-Oczko

**Affiliations:** 1 Oral Pathology Department, Faculty of Dentistry, Wroclaw Medical University, Ul. Krakowska 26, Wroclaw, 50-425 Poland; 2 Division of Clinical Pathology, Department of Clinical and Experimental Pathology, Wroclaw Medical University, Ul. Borowska 213, Wroclaw, Poland; 3 Section of Pathology, Department of Biomedical Sciences, University of Cagliari, Cittadella Universitaria - Monserrato, Monserrato, Italy

**Keywords:** Lichen Planus, Oral, Leukoplakia, Oral, Herpesvirus 1, Human, HPV, Oncogenic viruses

## Abstract

**Background:**

The association of *Human Papilloma Virus* (HPV) and *Human Syncytial Virus* (HSV) infection with inflammatory and potentially malignant disorders of the oral cavity (OPMD) is unknown. The aim of this cross-sectional study was to stablish the expression of the p16^INK4A^ and HSV proteins, to test potential correlation between those parameters in biopsies from clinically diagnosed oral lesions.

**Methods:**

Immunochemical analysis of 211 formalin-fixed, paraffin-embedded (FFPE) blocks from 211 individuals was provided. The clinical diagnosis included in the research were Oral lichen planus (*N* = 30), Oral Leukoplakia (*N* = 13) Mucocele (*N* = 25), Erosion/ulceration/ inflammation of mucosa (*N* = 8), Overgrowth of mucosa (*N* = 135).

**Results:**

Two hundred eleven analyzed FFPE samples resulted with the median age of 58.5 years (the average age 54.0 years and SD ± 17 years). The female/male ratio was 2.3 (69.7% vs 30.3% respectively). All the samples positive for HSV also expressed p16^INK4A^ (*p* = 0.000), that’s showed various levels of association with the diverse clinical diagnosis reaching the higher level in OM 49.1% (29 positive samples) and OLP 30.5% (18). p16^INK4A^ was associated with OLP at 30.5% (18), and fibroma 30.5%. HSV expression was mostly present in fibroma at 47.6% (10 positive samples).

**Conclusion:**

HSV and p16^INK4A^ positivity in relation to diagnosis of the biopsies showed statistically most often p16^INK4A^ in OLP and fibroma. The results of co-expression of p16^INK4A^ and HSV in mucocele and fibroma in oral mucosa suggest a cooperation between the molecular alterations induced by these two viruses. Squamous papilloma samples positive for p16^INK4A^ were also positive for HSV, suggesting that the putative pro-oncogenic action of HSV could be an early event.

**Supplementary Information:**

The online version contains supplementary material available at 10.1186/s12903-024-04105-z.

## Background

Oral squamous cell carcinoma (OSCC) is a disease caused by multitude of factors from which the confirmed and most discussed are tobacco smoking and alcohol drinking [1–3]. Additionally, as presented in the recent special report of International Agency for Research on Cancer entitled “IARC Perspective on Oral Cancer Prevention”, approximately only 2% of oral cancer worldwide is caused by Human papillomavirus (HPV) infection, primarily type HPV16 [4]. Indeed, high—risk human papillomaviruses (HR-HPV) type 16 and 18 are the most prevalent in potentially malignant lesions and squamous cell carcinoma [5–10]. On the other hand, there are low risk types confirmed correlations the oral squamous epithelium as in the case of *Verruca vulgaris* (2, 4 subtypes), squamous papilloma (6, 11 subtypes), morbus Heck’s (subtypes 13, 32), *condyloma acuminatum* (HPV subtypes 6,11).

A widely used surrogate marker for the screening for the presence of HPV is the p16^INK4A^ protein sometimes referred to as p16, which is overexpressed in precursor lesions associated with HR-HPV subtypes [11, 12]. It has been reported that in tumor tissue, HPV infection is related to the p16^INK4A^ protein expression [12]. Molecular mechanism of HPV involved in potential carcinogenesis is based on inhibiting Rb which leads to a high expression of p16^INK4A^ protein due to negative feedback regulation [13, 14]. In reference to Oral Squamous Cell Carcinomas (OSCCs), those processes have demonstrated overexpression of p16^INK4A^, as for what is typically observed in cervical intraepithelial neoplasia and cervical cancer [14]. However, as reported by Rosa et al., there are conflicting results in studies evaluating p16^INK4A^ expression in oral potentially malignant disorders (OPMDs), which may be due to the complexity of the mechanism involved [14], however the overexpression of p16^INK4a^ is indicative of HPV infection [15, 16].

Coexisting in the oral cavity HSV-1 is an epitheliotropic human pathogen potentially affecting half of the population [17] which once infecting the cell remains latent in the sensory ganglia of peripheral nerves, and can be reactivated under the cause of stress [18, 19]. This process might lead to secondary infection in oral epithelial cells [19]. In the infected area it has a neurotropic affinity, with rapid replication cycle transmitted through skin or mucosa, during childbirth, by infected body fluids (saliva, genital fluids, exudatives of active lesions), and causes most of primary infections of orofacial region and central nervous system [20, 21].

The effect of the concomitant presence of HPV and HSV-1 on risk of oral carcinoma has long been investigated by several epidemiological studies with inconsistent results [18, 19, 22]. The prevalence of HPV and HSV infection and the association with inflammation and OPMDs is uncertain. Oral lichen planus (OLP), and oral leukoplakia (OL) are forming a part of the OPMDs according to WHO classification [23, 24]. Although the etiology and risk factors leading to the progression of most common OPMD – leukoplakia – into OSCC is smoking and chewing tobacco, for oral lichen planus as a the chronic T-cell-mediated autoimmune disease this search is still ongoing [25, 26]. Among some of the factors named are the genetic background, type of dental materials used causing potential lichenoid reaction to it (as amalgam, metals, gold, and composite restorations) [25, 27], drugs taken by the patient (i.e., cardiovascular agents, non-steroidal anti-inflammatory drugs, hypoglycemics) [27, 28], and infectious agents like HSV and HPV [23]. In the reference to the OLP relation to both of the viruses, one of recent systematic reviews presented interesting relation to HPV [29]. Authors suggested that HPV infection may be an antigenic stimulus of cytotoxic T-lymphocyte expansion that characterizes severe erosive OLP [29, 30]. One study, although conducted on small number of subjects, presented that with the possible “hit and run” mechanism included, HSV-1 and HPV-16 type play a synergistic role in the development of oral cancer underlining that the HSV might be more frequently found in OPMDs than in carcinoma [22].

Co-expression of the p16^INK4A^ as a possible surrogate marker for HPV and HSV proteins in material collected from the oral cavity, especially in OPMDs, is a research matter not stressed enough in the current literature. Although, there is a growing body of evidence pointing that the p16^INK4A^ over-expression does not unequivocally represent the active transcription of HPV in these disorders underlining the possibility of other mechanisms related to cell cycle and molecular pathways that may fuel the positive immunohistochemical staining for p16^INK4a^ [16].

Since the infection with HSV and HPV represents the global burden [31], the aim of this study was to establish the prevalence of p16^INK4A^ surrogate marker for HPV and HSV in biopsies with the different clinical and histopathological diagnosis, taken from diverse oral mucosa sites, among them OPMD’s.

## Methods

### Material and study group

In this retrospective cross-sectional study on archival 211 formalin fixed paraffin-embedded (FFPE) tissue samples of different oral mucosa pathologies were assessed. Individuals’ biomaterial was collected at Dental Polyclinic in an urban area by a group of dentists. Included material was selected from 2 years span, and collected within 6 months by a medical professional. Inclusion criteria for the archival biomaterial selection from the Department of Pathomorphological and Oncological Cytology was benign and OPMDs along with potentially malignant lesions that were fully diagnosed by dental professionals and received the pathomorphological diagnostic result. Exclusion criteria were: 1) receiving the OSCC diagnosis along with other cancer diagnostics and 2) incomplete medical and dental history or no histopathological result included. Clinical data was included along with the epidemiologic data of the individuals. The quality of the FFPE blocks was evaluated and only after the sufficient amount of the material was present the evaluation was performed.

In the study general information like gender, age, location of the lesion on the oral mucosa, its clinical and pathomorphological diagnosis, immunological prevalence of p16^INK4A^ as a surrogate marker for HPV and HSV were assessed. The study was approved by the Research Ethic Committee of Wroclaw Medical University – number KB-320/2020.

### Methodology

#### Immunohistochemical staining (IHC) and antibodies used in research

Tissue samples were fixed in 10% buffered formalin, dehydratated and embedded in paraffin. Hematoxylin and eosin-stained (H&E) preparations were done on all the samples. The IHC reactions were performed on 4-µm-thick paraffin sections fixed to microscopic slides (SuperFrost + , Menzel Glässer, Braunschweig, Germany). Deparaffinization and antigen retrieval were performed in Target Retrieval Solution, pH 9 (97^o^ C, 20 min) and a PT Link Rinse Station like in our previous research [32]. The sections were then washed in TBS and incubated with primary antibody (RT, 20 min) in Link48 Autostainer. The following primary antibody was used: monoclonal anti- p16^INK4A^, (clone E6H4, Dako) and rabbit polyclonal anti-HSV-1 antibody ab9533 (Abcam, USA). EnVison FLEX (DakoCytomation) reagents were used for visualization of the studied antigen and the slides were counterstained with haematoxylin, as recommended by the manufacturer. The IHC reactions were accompanied by the standardized controls as per manufacturers requirements and instructions.

#### Interpretation of immunohistochemical staining

Both p16^INK4A^ and HSV immunohistochemical diagnosis was performed by an experienced certified pathologist, head of the Department of Pathomorphological and Oncological Cytology (A.H.), but the intensity of the immunohistochemical reaction was estimated independently by two pathologists as described in our previous work [33]. Tissue specimens were histologically verified to confirm the diagnosis, and histological type and the diagnosis was performed after the verification of Hematoxylin – Eosin staining. An Olympus BX43F light microscope (Olympus America, Inc., Melville, NY, USA) was used for the evaluation of slides.

Proteins expression in tissues was assessed semi quantitatively, considering the intensity of immunostaining and the number of cells showing immunoreactivity for the analyzed proteins. The expression of p16^INK4A^ for HPV and HSV proteins was evaluated in FFPE samples according to a modified Remmele scale [34, 35] by combining the intensity of staining and pattern of distribution. Expression of p16^INK4A^ proteins was graded based on reaction intensity juxtaposed with the staining intensity of positive controls. The evaluation included 2 parameters of protein expression:

1. Two types of pattern distribution in expressed results:
  Focal,
  Diffused.
2. Intensity of staining:
  Int 1 = weak,
  Int 2 = moderate,
  Int 3 = strong.

### Statistical method

For each parameter mean (X), median (M), standard deviation (SD, range (min, max), lower and upper quartile (25Q, 75Q) were calculated. Statistical significance between means for different groups was calculated by the non-parametrical U Mann–Whitney test (for two groups) or Kruskal–Wallis test (for more than two groups). The homogeneity of variance was determined by the Levene’s test. Statistical significance between frequencies was calculated by the chi-square test χ^2^_df_ with corresponding degree of freedom df (df = (m-1)*(n-1), where m – number of rows, n – number of columns). A *p* value of less than 0,05 was required to reject the null hypothesis. Statistical analysis was performed using EPIINFO Ver. 7.2.4.0 and Statistica Ver. 13.3. software packages.

## Results

### Individuals’ characteristics

Investigated specimens were retrieved from 211 individuals with the median age of 58.5 years (the average age 54.0 years and SD ± 17 years). The female/male ratio was 2.3 (69.7% vs 30.3% respectively).

During the selection process archival biomaterial (FFPE samples), individuals clinically evaluated in the Oral Pathology Outpatient Clinics representing changes on the oral cavity were included. Search through the archival biobank material was based on positive histopathological evaluation for benign and potentially malignant OPMDs, including all grading of the samples from the histopathological evaluation point (from simple keratosis, hyperplasia to stage of oral lichen planus, fibroma, mucocele, epithelial hypertrophy/ hyperplasia, inflammatory infiltration, ulceration and squamous papilloma), and clinical indications referring to the visual changes on the oral mucosa. Whenever the clinical evaluation of the change on the oral mucosa was provided, it was presented by the dentist in medical referral to the histopathology diagnostics, in order to evaluate its potential malignancy. Specific division of those samples was presented in Table S2 and Table 3. Possibly some of the fibromas might have been caused by irritation of the dental prosthesis. Secondly, as commonly preferred, if patients arrives to Oral Pathology clinic with aphthous lesions, then it is firstly directed for the topical/general treatment. If the aphthous change is persistent, after changing into persistent ulceration with potentially risky background is diagnosed by biopsy. Terminology of erosion was used for the erosive, not ulcerative changes on the oral mucosa that however were put as the same group for the statistical analysis because of the potential inflammatory background of those disorders. In the Oral Pathology Department patients with ongoing herpetic rash on the lip or in the oral cavity, representing the HSV infection, are asked to postpone the visit. For this reason, the ulcerations present were not related to the ongoing HSV process.

### p16^INK4A^ -HPV and HSV diagnostics

p16^INK4A^ was detected in 59 individuals (28%), most of which were females (74.6%). The median age was 59.0 years. From a pathological point of view, the pattern of distribution of p16^INK4A^ positivity was predominantly focal (61%) and individuals with this pattern were predominantly younger females than those with the diffuse pattern. The semiquantitative analysis of IHC revealed a medium intensity of the stain in 42.4% of the individuals, a low intensity in 39%. The intensity was high only in 18.6% of cases.

Unlike p16^INK4A^, the HSV expression was observed with a low prevalence, only in 21 individuals – 9.95%, and similarly to p16^INK4A^ with higher frequency in female subgroup. The focal pattern of HSV distribution was detected more often—in 62% of all specimens, and in the lower individuals age of average 53 years. HSV expression intensity in all the 21 positive samples was observed in the low level described as “Int 1”, in medium level described as “Int 2”, and there was no high intensity described as “Int 3”. In the male subgroup there was present only low intensity expression described as “Int 1” in connection with the younger medium age of 53 years (Table 1). In this table, the Kruskal–Wallis test analysis was evaluated to illustrate the relation between different expressions of HPV and HSV with the age, and subsequently Fisher exact test and was evaluated to illustrate the relation between gender of the included subjects.

**Table 1:** Distribution and types of p16^INK4A^ and HSV expression in relation with the subjects age and gender

*Investigated specimens*	*Study group* *Total* = *211* *Median age* *(years) (25Q ÷ 75Q)*	*P* *age*	*Female* *Total* = *147*	*Male* *Total* = *64*	*P* *gender*
*p16*^*INK4A*^* expression intensity*					
*Int 1 – 23 P (39%)*	53.0 (46.0 ÷ 59.0)	*p* = 0.0024	16 (10.9%)	7 (10.9%)	*p* = 0.700 χ^2^_2_ = 0.71
*Int 2 – 25 P (42.4%)*	61,0 (48.0 ÷ 68.0) 65,0 (62.0 ÷ 71.0)		20 (13.6%)	5 (7.8%)	
*Int 3 – 11 P (18.6%)*			8 (5.4%)	3 (4.7%)	
*HSV expression intensity*					
*Int 1—19 (90.5%)*	53 60		14 (9.5%) 2	5 (7.8%) 0.0	*p* = 0.999
*Int 2—2 (9.5%)*					
*Int 3—0 specimens*					

**Fig. 1:**
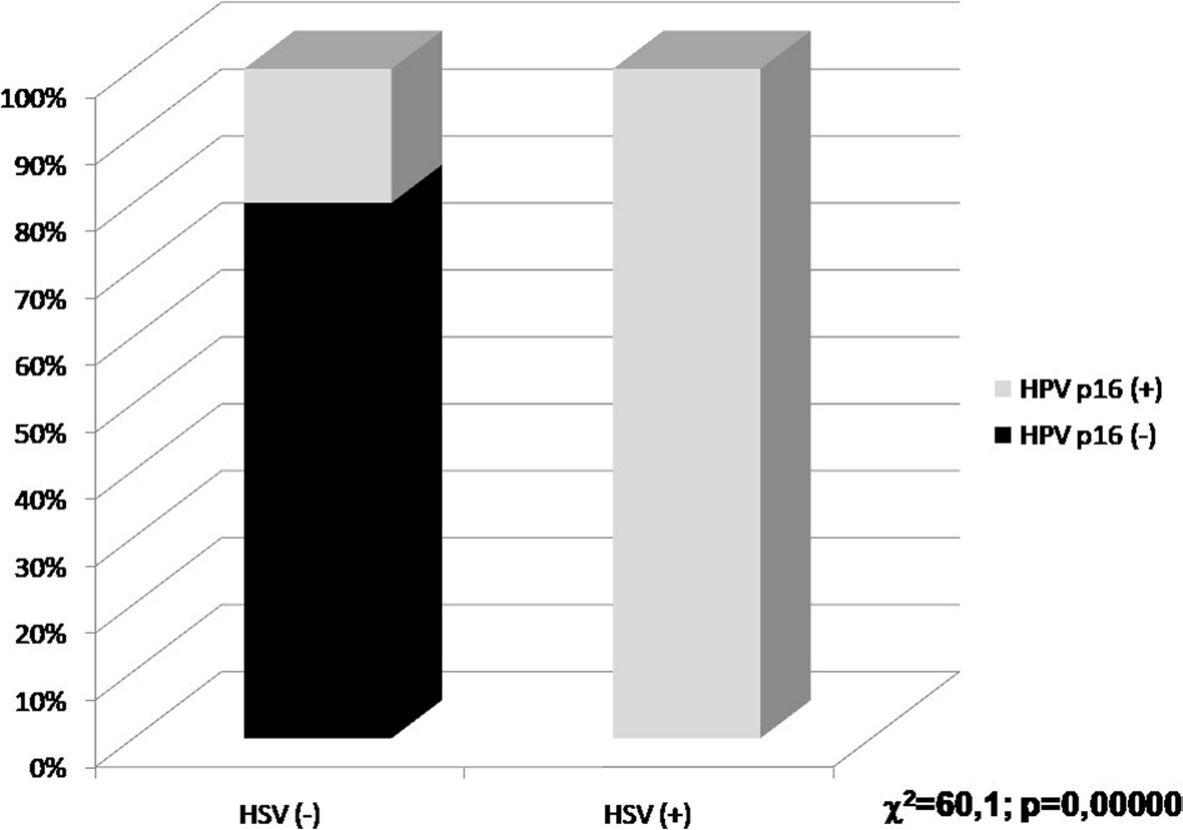
Correlation between the p16^INK4A^ protein for HPV and HSV expression

Statistically important strong correlation was found between the p16^INK4A^ and HSV expression. There was no detected isolated HSV positivity, and in all the samples with HSV positivity also HPV p16^INK4A^ was expressed (*p* = 0.000), as presented in the Fig. 1. There was also correlation between the HPV and HSV expression patterns (*p* = 0.000).

### HPV and HSV expression of the oral sites and clinical diagnosis

**Fig. 2:**
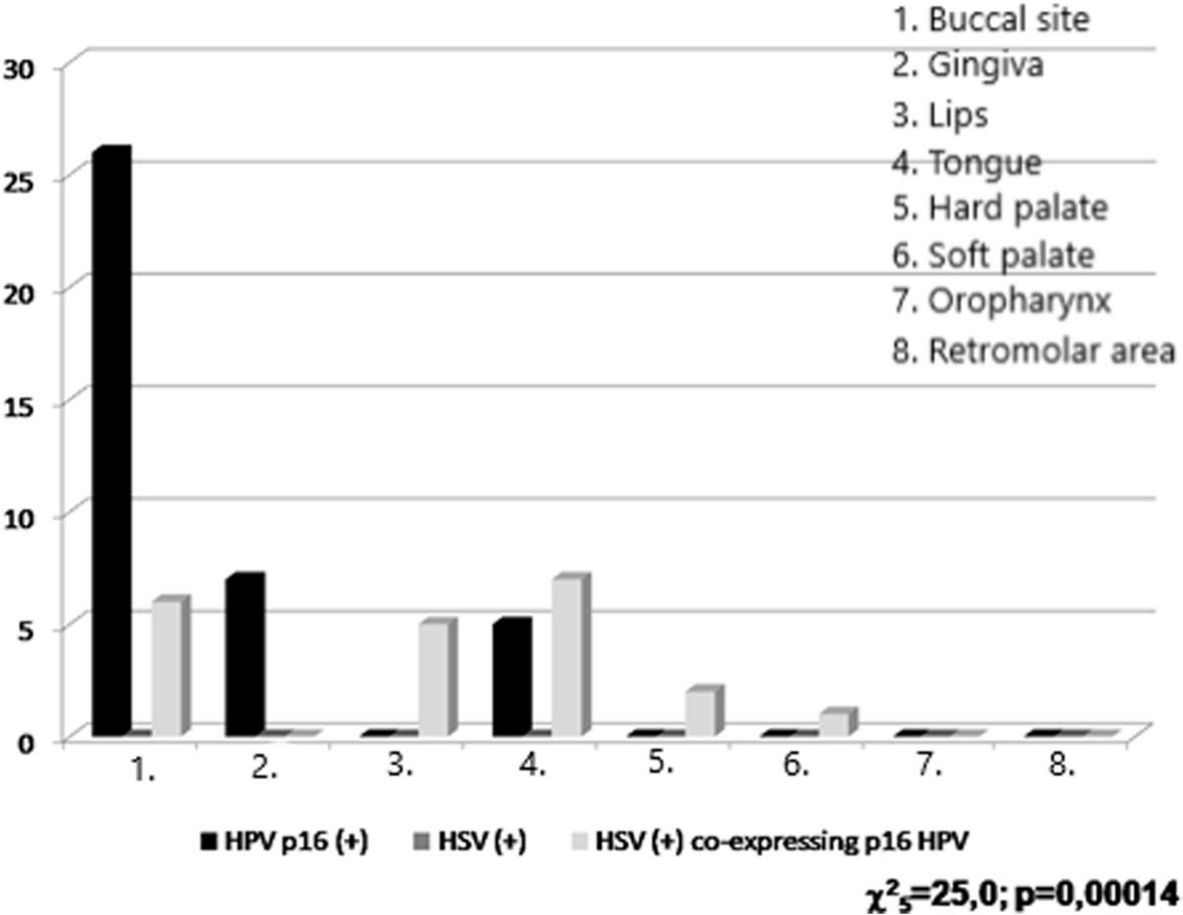
p16^INK4A^ and HSV expression presence in connection with the site of oral cavity

The antibody p16^INK4A^ was statistically most often detected in the specimens from buccal sites -54.24%, (*p* = 0.005%). HSV were most often expressed in samples from tongue—33.3%, but without statistical significance. All clinical sites evaluated are included in Fig. 2.

In the estimation of p16^INK4A^ and HSV positivity in connection with the clinical diagnosis, statistical difference was shown among the overgrowth of mucosa samples (*p* = 0.000) (Table 2). Percentage in the Table 2 were established reference to total number of clinical diagnosis within the group (oral lichen planus, leukoplakia, mucocele, erosion and overgrowth).

**Table 2:** p16^INK4A^ and HSV expression presence in connection with clinical diagnosis

*Clinical diagnosis*	*Number of specimens*	*p16*^*INK4A*^* expression among the oral lesion (59 total positive)*	*HSV expression (21 total positive)*	*HSV* + *samples co-expressing HPV*
*Oral lichen planus*	30	18 (60.0% OLP)	1 (3.33%)	1 (3.33%)
*Leukoplakia*	13	7 (53.9% OL)	2 (15.4%)	2 (15.4%)
*Mucocele*	25	4 (16.0% MUC)	3 (14.3%)	3 (14.3%)
*Erosion/ ulceration/ inflammation of mucosa*	8	1 (12.5% EUI)	0 (0.0%)	0 (0.0%)
*Overgrowth of mucosa*	135	29 (21.5% OVG)	15 (11.1%)	15 (11.1%)

χ^2^_4_ = 25,2 *p* = 0,00005 for p16^INK4A^ expression

χ^2^_8_ = 31,1 *p* = 0,00014 for the HSV expression

*OLP* oral lichen planus, *OL* oral leukoplakia, *MUC* mucocele, *EUI* Erosion/ ulceration/ inflammation of mucosa, *OVG* overgrowth of mucosa

**Fig. 3:**
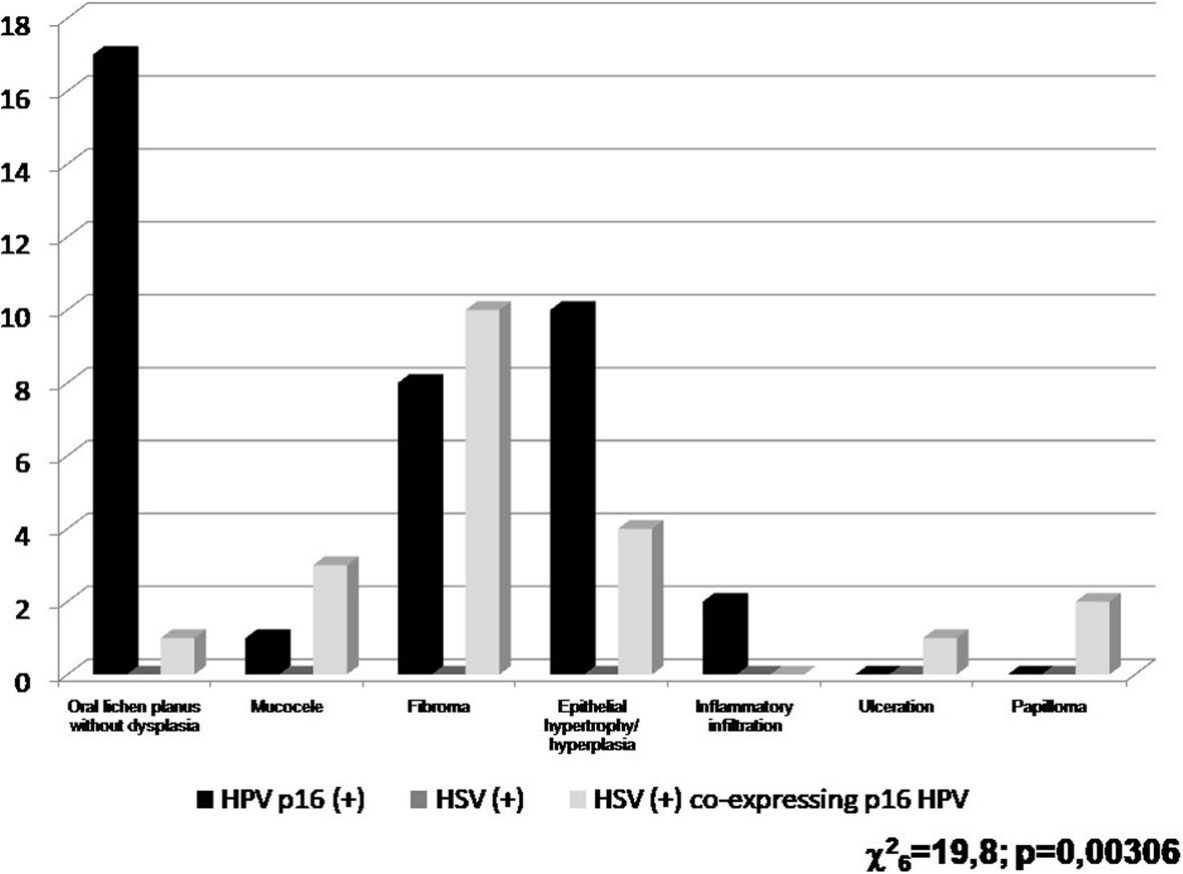
Summary of p16^INK4A^ and HSV detection in connection with histopathological diagnosis

The examination of both the HSV and p16^INK4A^ positivity in relation to histopathological diagnosis of the biopsies showed statistically most often p16^INK4A^ in OLP and fibroma of 30.5% in both types of samples (*p* = 0.000) in reference to total positive number HPV samples. HSV expression instead, was most often present in fibroma in 19.1% from whole HSV positive samples (Table 3, and Fig. 3 for the comparison). Whole number of evaluated samples either those negative for p16^INK4A^ and HSV were included for transparency. Percentage in the Table 3 were established on the basis of positive expressions resulted in the group of same histopathologically analyzed samples (oral lichen planus, mucocele, fibroma etc.). Percentage in the Table 3 were established on the basis of total positive expressions resulted in the group (p16^INK4A^ and HSV positive).

**Table 3:** p16^INK4A^ and HSV detection in connection with histopathological diagnosis

*Number of histopathological diagnoses*	*Number of specimens*	*p16*^*INK4A*^* expression for HPV among the oral lesion (59 total positive)*	*HSV expression among the oral lesion (21 total positive)*
*Oral lichen planus*	29	18 (62.07% OLP)	1 (3.5% OLP)
*Fibroma*	81	18 (22.2% FIB)	10 (12.4% FIB)
*Mucocele*	20	4 (20.0% MUC)	3 (15.0% MUC)
*Epithelial hypertrophy/ hyperplasia*	34	14 (41.2% EHH)	4 (11.8% EHH)
*Inflammatory infiltration*	16	2 (12.5% II)	0 (0.0% II)
*Ulceration*	6	1(16.7% U)	1 (16.7% U)
*Papilloma*	21	2 (9.52% P)	2 (9.5% P)
*Others*	4	0 (0.0%)	0 (0.0%)

Evaluation of all the specimens from different sites of oral cavity showed that the pattern of p16^INK4A^ distribution was more often focal than diffused in specimens from buccal site, gingiva, and lips, in turn in the samples from tongue diffuse pattern was two times more often detected 21.0% vs 10.5%. In the samples from gingiva the most often present was the highest intensity of HPV-related p16^INK4A^ positivity at the level of 27.3%. These differences were of statistical importance (*p* = 0.017). In turn the HSV expression pattern was more often focal in buccal site, lips, and tongue. The local and diffuse pattern of distribution was on the same level of 12.5% in the biopsies from the hard palate but there were only 2 positive HSV specimens. All the data about the site of specimen evaluation is available in the supplementary data table (Table nr S1).

Evaluation of all the specimens with the different clinical diagnoses showed that in mucocele and overgrowth of mucosa the local pattern of p16^INK4A^ distribution was more often present, whereas in OLP both distribution patters were present in the same percentage (30.0%). The presence of the highest intensity of expression was not detected in mucocele and samples categorized as erosion, ulceration, and inflammation (*p* = 0.000). On the other hand, the HSV focal pattern of inflammation was more often seen than diffused pattern in OLP biopsy, mucocele and samples taken from mucosa with overgrowth, and in the leukoplakia both patterns were present in the same percentage (Table 4). In the HSV expression the high intensity described as was not present.

**Table 4:** Pattern and intensity of p16^INK4A^ and HSV expression in connection with most significant clinical diagnosis

Clinical diagnosis	*p16*^*INK4A*^* distribution pattern number of specimens*			*p16*^*INK4A*^* expression intensity number of specimens*			*HSV distribution pattern number of specimens*			*HSV expression intensity number of specimens*		
	Lack of p16^INK4A^ expression	Focal	Diffuse	Int 1	Int 2	Int 3	Lack of p16^INK4A^ expression	Focal	Diffuse	Int 1	Int 2	Int 3
Oral lichen planus *N* = 30	12 40.0%	9 30.0%	9 30.0%	3 10.0%	9 30.0%	6 20.0%	29 96.7%	1 3.3%	0 0.0%	1 3.3%	0 0.0%	0 0.0%
Leukoplakia *N* = 13	6 46.1	3 23.1%	4 30.8%	2 15.4%	3 23,1%	2 15.4%	11 84.6%	1 7.7%	1 7.7%	2 10.5%	0 0.0%	0 0.0%
Overgrowth of mucosa *N* = 135	106 78.5%	21 15.6%	8 5.9%	15 11.1%	11 8.1%	3 2.22%	120 88.9%	9 6.7%	6 4.4%	14 10.4%	1 1.0%	0 0.0%

### HPV and HSV expression of the oral sites and histopathological diagnosis

In the reference to the co-expression of the p16^INK4A^ related with HPV with the HSV protein on the oral site, the highest percentage of the co-expressed samples were in the tongue region. Secondly, 28.6% represented the cheek localization, and thirdly lip as presented in the Table 5.

**Table 5:** Oral site and p16^INK4A^ and HSV co-expression

*Oral cavity localisation*	*p16*^*INK4A*^ +	*p16*^*INK4A*^ + *and HSV* + *(co-expression)*	*Total*
*Cheek*	**26**	**6**	32
	68,42%	28,57%	
*Lip*	**0**	**5**	5
	0,00%	23,81%	
*Tongue*	**5**	**7**	12
	13,16%	33,33%	
*Gingiva*	**7**	**0**	7
	18,42%	0,00%	
*Hard palate*	**0**	**2**	2
	0,00%	9,52%	
*Soft palate (oropharynx)*	**0**	**1**	1
	0,00%	4,76%	
*Summary*	**38**	**21**	59

In the assessment of the all the specimens regarding different histopathological diagnosis the focal pattern of p16^INK4A^ distribution was more often present in fibroma, in mucocele, in epithelial hypertrophy/hyperplasia and ulceration in comparison to the diffuse pattern distribution.

**Fig. 4:**
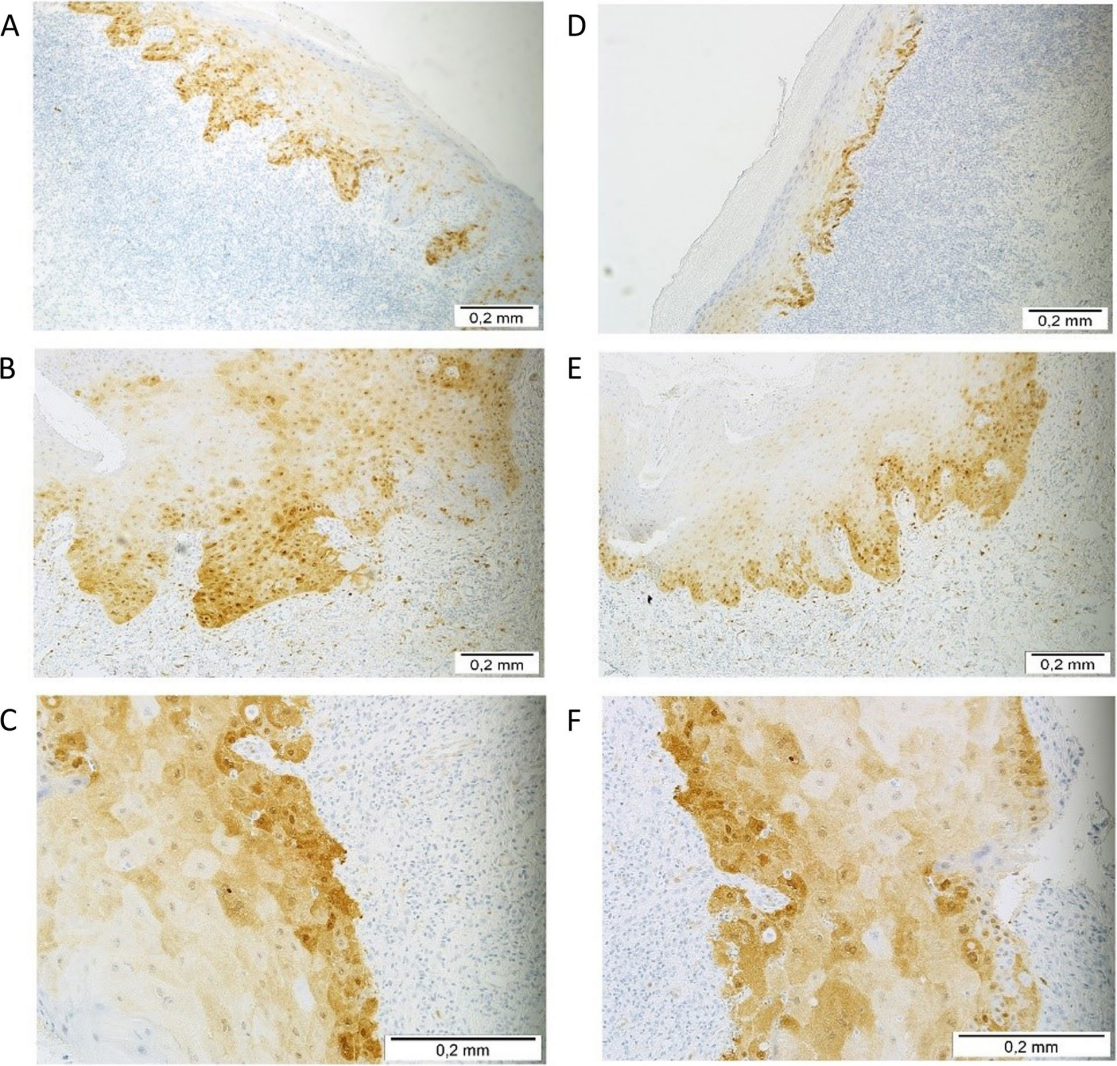
Representative immunostaining of p16^INK4A^, and presence of HSV in OLP and OL cases. **A** p16^INK4A^ immunostaining observed in epithelial cells of OLP; **B** p16^INK4A^ immunostaining observed in a high percentage of the OL tissue; **C** p16^INK4A^ protein expression OLP; **D** HSV expression in the OLP tissue; **E** HSV expression in OL; **F** HSV protein expression OLP (EnVision technique). The scale refers to the 0.2 mm in real dimensions

The samples with the OLP histological diagnosis presented both focal and diffuse pattern of p16^INK4A^ in the same percentage (both representing 31%). The highest intensity of p16^INK4A^ positivity was not detected in mucocele, similarly to clinical diagnosis of this pathology and in inflammatory infiltration, ulceration, papilloma. The representation of immunostaining process on the OLP and OL FFPE samples are shown in the Fig. 4.

**Fig. 5:**
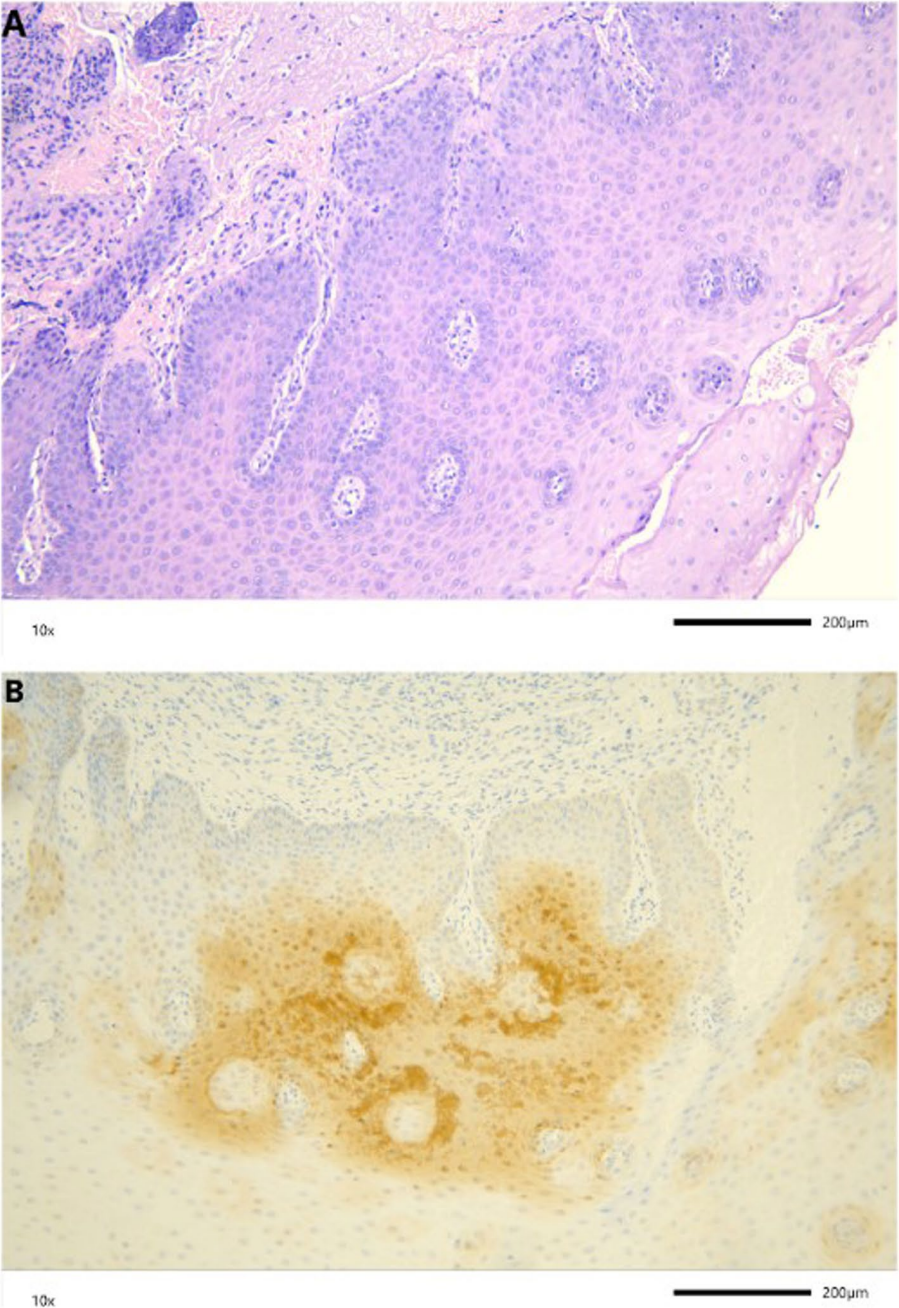
**A** Hematoxylin and eosin slide observed in epithelial cells of oral mucosa, **B** Representative immunostaining of HSV protein expression, representation of diffused pattern of moderate intensity. The scale refers to the 0.2 mm in real dimensions

**Fig. 6:**
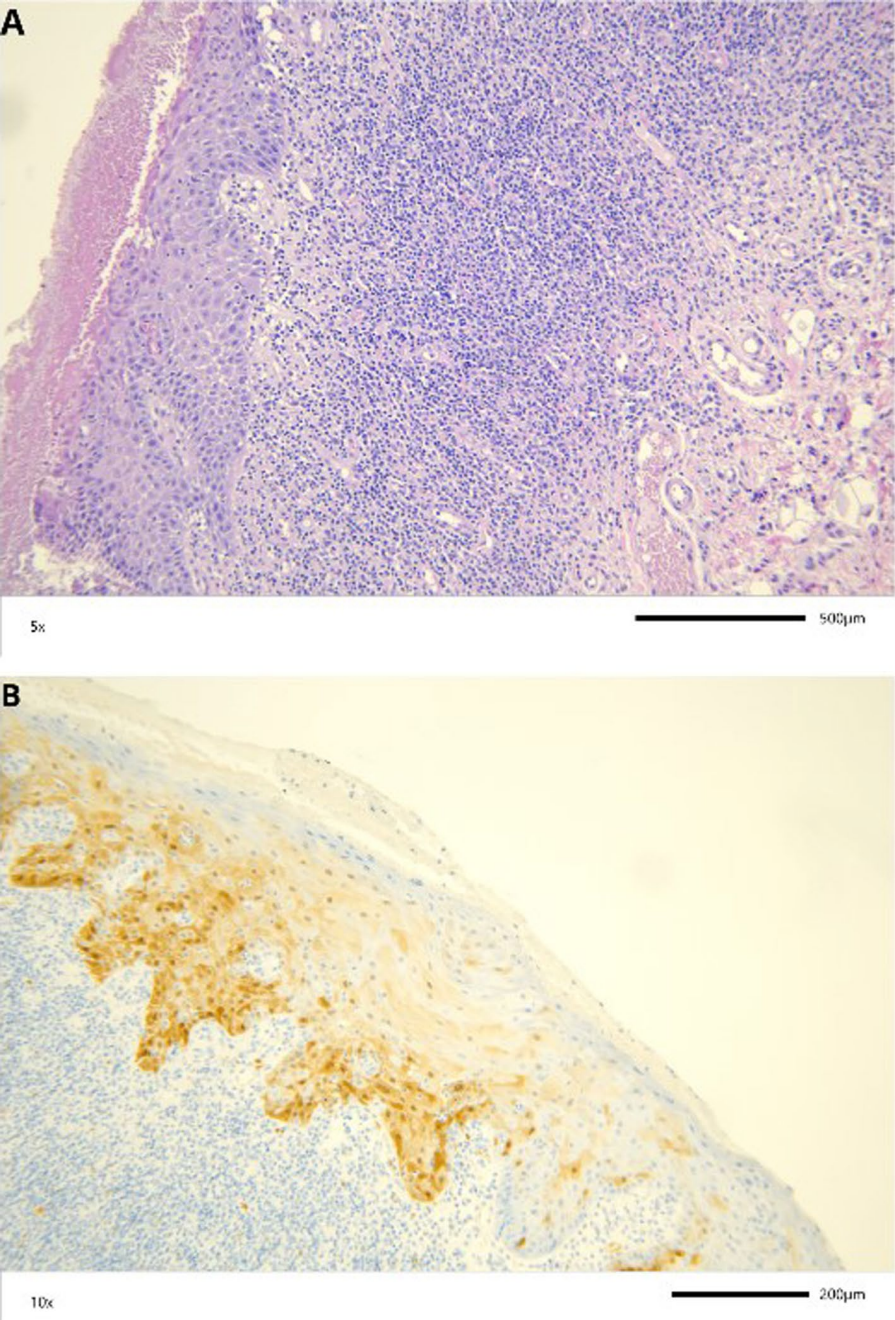
**A** Hematoxylin and eosin slide observed in epithelial cells of OLP; **B** Representative immunostaining of p16^INK4A^ protein expression, representation of diffused pattern of moderate intensity in OLP. The scale refers to the 0.2 mm in real dimensions

In order to represent the Hematoxylin–Eosin and Immunohistochemical staining of two subjects from different pathologies of the oral mucosa, two representative sets of images were taken with the use of Leica Microsystems CMS GmbH, Model DM2000 LED, and Color Digital Microscope Camera Jenoptik progress Gryphax (Figs. 5 and 6). Each one of the images represent the same subject.

## Discussion

Here analyzed data included the archival FFPE samples diagnosed in different grades of oral mucosa pathologies as oral lichen planus, oral leukoplakia, mucocele, clinically diagnosed by a dentist erosion/ ulceration/ inflammation of mucosa and the overgrowth of the oral mucosa. Use of the p16^INK4A^ protein was provided as a surrogate marker for HPV and to evaluate the co-expression of another important for the oral health virus, the HSV staining was evaluated. In reference to the HPV surrogate marker p16, the results display the highest expression frequency from biopsies on buccal site (54% of positive results) and tongue (20% of positive results) as presented in supplementary data. These results are in concordance with D’Souza et al., also stating that tongue was constituting majority of HPV-associated head and neck squamous cell carcinomas localizations in their research [36]. Regarding clinical diagnosis, the highest level of expression was shown in samples of OLP (60.0% of clinically diagnosed samples resulting positive for p16^INK4A^ related to HPV expression) and OL (53.9% of oral leukoplakia diagnosed samples), then mucosa overgrowth (21.5% of individuals with the clinical diagnosis) and mucocele (16% accordingly). In the research of Rosa et al., 20.65% of cells observed in OLP lesions were positive for p16^INK4A^, also suggesting that HPV may be present in OLP [14]. In another research analyzing 103 samples of the OL group, by Li-Qun Yang et al., p16^INK4A^ rate for HPV was 4.9% (5/103) [37]. All HPV-positive OL cases had p16^INK4A^ overexpression, however as stated by the researchers the sensitivity of p16^INK4A^ histopathological diagnosis was considered poor. In the analyzed study, the 88.4% of p16^INK4A^ over-expressed OL were HPV negative.

In our study p16^INK4A^ expression was detected only in 2 samples with pathological diagnosis of squamous papilloma (9.52% of all positive results). That seems to find an explanation in the molecular biology type of research, where even with the use of DNA diagnostics from the lesion, not in all cases viral genetic material is detected as stated by Syrjanen et al. [38]. Whenever potential false negative result for HPV is present in the malignancy that is clinically related to HPV, the “hit and run” scenario might be applied [13, 39]. It suggests that those from the viruses, that have an activating role in the cancer development may disappear after the host cell accumulates numerous mutations [39].

Squamous papilloma positive for p16^INK4A^ were also associated with positivity for HSV in our work. Additionally, statistically important strong correlation was found between the p16^INK4A^ and HSV expression, and there was no detected isolated HSV positivity – in all the samples with HSV positivity also HPV p16^INK4A^ was expressed. Strong correlation between HPV p16^INK4A^ and HSV pattern distribution was found. Firstly, there were no isolated HSV expressions, all the 21 cases were present in the same specimens parallelly with HPV p16^INK4A^ expression. As described before, both oral and genital HSV infections are considered to be predisposing factors for HPV infection [18]. Guidry and Scott suggested that infection with HSV allows better access for HPV to the basal cell layer of the tissue, what subsequently eases the infection process. Thus, the HSV replication in tissues where HPV also replicates may influence persistence, clearance, and/or oncogenic activities of HPV. During in-vitro procedures HSV has shown the potential to alter aspects of the HPV life cycle upon co-infection [18]. However, the data of the coinfection in the head and neck and oral region is inconsistent. As reported by the researchers from Turku, Finland, head and neck squamous cell carcinomas individuals not treated with chemoradiotherapy and co-infected with HSV-1 and HPV had a worse outcome [40]. No similar data is available on the pre-malignant and OPMDs.

In the estimation of p16^INK4A^ and HSV positivity in connection with the clinical diagnosis, statistical difference was shown among the overgrowth of mucosa samples when referring to the total number of positively diagnosed samples. Data from literature indicate the prevalence of HPV in the oral cavity ranging from 17.7% to 1.0% [31, 41]. As described by Paver et al. HPV positive squamous cell carcinoma arising in the head and neck region does not carry the favorable prognosis [42]. Less is known however about the risk it is carrying in OPMDs. Systematic review and meta-analysis of 66 studies analyzed by Tam et al. [43] described the overall oral HPV prevalence as 7.7% for all HPV types. The availability of the data increases with the severity of the disorder presented by individuals, and the diagnostics of OPMD for the biomarkers that might be used in the prevention, and early diagnostics of those disorders.

In our study in the investigated group of 211 individuals HSV expression prevalence was 9.95%, and the median age of 21 positive subjects was 53 years. This expression was more often present in female in the rate of 76.2%. The high intensity of HSV expression was not detected. This is however understandable, since all the cuts (biopsies) were performed in the dental studios by a dentist, where the rule applies that all the individuals with clinical representation of HSV are asked to postpone their visit due to biosecurity protocol to avoid contamination of the dental appliances during the dental procedures, and infection of the dental personnel [19]. HSV were most often expressed in samples from tongue, however without statistical significance. Higher expression on this part of the oral mucosa can be explained by the mode of transmission of the virus and its’ affinity for nerve cells, highly represented on the lips and tongue. The molecular mechanism of infection involves the latency and reactivation of the virus from its stronghold, the trigeminal ganglion [44]. Clinical representation of the HSV tongue infection is also commonly associated with acute primary herpetic gingivostomatitis and in recurrent infection on the tongue has been described in the immunocompromised individuals [45]. There is also report of an oral ulcer co-infection of HSV, cytomegalovirus (CMV), and Epstein-Barr virus (EBV) in a recipient of kidney-pancreas transplant [46]. However, in our study, no immunocompetent individuals were included, and the expression of the HSV was low.

There have been contradictory results discussed in the literature on the involvement of HSV-1 in oral carcinogenesis, that might be important in here presented study on OPMDs [47]. Latest research however presented that cell survival or invasion was not affected at low doses of HSV-1, when oral tongue squamous cell carcinoma was followed-up [47]. Further research should be performed in order to establish its role in the OPMDs to provide if similar pattern might be observed.

In the field of OLP diagnostics when the HSV is concerned there is little research discussed. Report on the presence of the human herpesviruses by ÓFlatharta, et al. provide the outcome of no correlation between this group of viruses being the causative agents for the OLP [48]. In the work provided by Cox et al., the HSV-1 presence was relatively small in the OLP group of individuals, and also the connection between the HPV and HSV in those samples were confirmed [22]. In the assessment of 60 biopsies of OSCC from Iranian individuals only three samples were positive indicating not important role of this expression in carcinoma development [49].

To our knowledge here presented is the first presentation of the prevalence of two proteins, both HPV- p16^INK4A^ and proteins characteristic for HSV expression, evaluated in the same samples of oral benign, and potentially malignant lesions. Also, the illustration of these expressions in connection with the site of oral cavity assessed, clinical and histopathological investigations and described distribution pattern and their intensity seem to give new approach to detection and diagnosis of such expressions. Findings about the higher frequency of the HPV- p16^INK4A^ in OLP patient’s biomaterial is in concordance with the general line of research [40], but more prospective cohort studies are needed to establish the impact of the HPV prevalence among OLP individuals on the possible transformation to more severe oral lesions, and OSCC. In conclusion, further studies are required to determine the possible role of viral co-infections with HPV and HSV-1 as risk markers for the development of OSCC from OPMDs [50].

HPV p16^INK4A^ is not treated as a gold standard for the oncogenic HPV expression, which is estimated to be E6/E7 mRNA in situ hybridization [51]. Here discussed work was proposed in order to use the clinically accepted and widely used biomarker as p16^INK4A^ with the acceptance of its limitations. Present study wasn’t aiming to investigate the molecular diagnostics of the lesions, since the mRNA from FFPE samples might be unstable, and such a decision could devaluate the number of cases collected for this study. In here present research, the effect of the proportion size of lesions is to be considered in the statistical analysis of the data, as the study was not planned in order to pair by the type of lesion. Non-pairing of the number of lesions but by the continuity of the clinical performance could create a sampling bias important in the statistical analysis. Additionally, the authors report the lack of information about the general health of the investigated individuals in here present study, but researchers were not evaluating the progression of the lesions, nor survival rate because of the exclusion of OSCC samples from the analysis.

## Conclusions

In conclusion, the presented data suggests a possible correlation between the expression of HPV- p16^INK4A^ and HSV in oral mucosa of individuals with different types of OPMD. The examination of the HSV and p16^INK4A^ positivity in relation to diagnosis of the biopsies showed statistically most often p16^INK4A^ in OLP and fibroma in both types of samples. The highest prevalence of double positivity was found in mucocele and fibroma, what suggest a cooperation between the molecular alterations induced by these two viruses. Squamous papilloma samples positive for p16^INK4A^ were also positive for HSV, suggesting that the putative pro-oncogenic action of HSV could be an early event.

### Supplementary Information

**Supplementary Material 1.**

**Supplementary Material 2.**

## Data Availability

The datasets supporting the conclusions of this article are included within the article and its additional supplementary files.
