# Supplementary material for: HPV related p16INK4A and HSV in benign and potentially malignant oral mucosa pathologies
Source: BMC Oral Health. 2024 Mar 18;24:347. doi: 10.1186/s12903-024-04105-z (PMC10949823; doi:10.1186/s12903-024-04105-z)
Supplement: Supplementary file 1 — Supplementary Material 1. [file 12903_2024_4105_MOESM1_ESM.docx]

**Table S1.** Pattern and intensity of p16^INK4A^ and HSV expression in different sites of oral cavity.

| Site of the biopsy specimens  Number of specimens | p16^INK4A^ pattern number of specimens | | | p16^INK4A^ intensity /number of specimens | | | HSV pattern number of specimens | | | HSV intensity /number of specimens | | |
| --- | --- | --- | --- | --- | --- | --- | --- | --- | --- | --- | --- | --- |
|  | Lack of expression | Focal | Diffuse | Int 1 | Int 2 | Int 3 | Lack of iexpression | Focal | Diffuse | Int 1 | Int 2 | Int 3 |
| Buccal site  N=70 | 38  54.3% | 21  30.0% | 11  15.7% | 11  15.7% | 15  21.4% | 6  8.6% | 64  91.4% | 4  5.7% | 2  2.9% | 6  8.6% | 0  0.0% | 0  0.0% |
| Gingiva  N=46 | 39  84.8% | 6  13.0% | 1  2.2% | 2  8.7% | 2  8.0% | 3  27.3% | 46  100.0% | 0  0.0% | 0  0.0% | 0  0.0% | 0  0.0% | 0  0.0% |
| Lips  N=43 | 38  84.4% | 4  8.9% | 1  6.7% | 2  4.6% | 3  7.0% | 0 | 38  84.4% | 4  8.9% | 1  6.7% | 4  8.9% | 1  6.7% | 0  0.0% |
| Tongue  N=38 | 26  68.4% | 4  10.5% | 8  21.0% | 6  15.8% | 4  10.5% | 2  5.3% | 31  81.6% | 4  10.5% | 3  7.9% | 6  15.8% | 1  2.63% | 0  0.0% |
| Hard palate  N=8 | 6  75.0% | 1  12.5% | 1  12.5% | 2  25.0% | 0 | 0 | 6  75.0% | 1  12.5% | 1  12.5% | 2  25.0% | 0  0.0% | 0  0.0% |
| Soft palate  (oropharynx)  N=3 | 2  66.7% | 0  0.00% | 1  33.3% | 0 | 1  33.3% | 0 | 2  66.7% | 0 | 1  33.3% | 1  33.3% | 0  0.0% | 0  0.0% |
| Bottom of the mouth  (oropharynx)  N=2 | 2  100.0% | 0  0.0% | 0  0.0% | 0  0.0% | 0  0.0% | 0  0.0% | 2  100.0% | 0  0.0% | 0  0.0% | 0  0.0% | 0  0.0% | 0  0.0% |
| Retromolar site  n=1 | 1  100% | 0  0.0% | 0  0.0% | 0  0.0% | 0  0.0% | 0  0.0% | 1  100.0% | 0  0.0% | 0  0.0% | 0  0.0% | 0  0.0% | 0  0.0% |
