# Supplementary material for: HPV related p16INK4A and HSV in benign and potentially malignant oral mucosa pathologies
Source: BMC Oral Health. 2024 Mar 18;24:347. doi: 10.1186/s12903-024-04105-z (PMC10949823; doi:10.1186/s12903-024-04105-z)
Supplement: Supplementary file 2 — Supplementary Material 2. [file 12903_2024_4105_MOESM2_ESM.docx]

**Table S2.** Pattern and intensity of p16^INK4A^ and HSV expression in connection with histopathological diagnosis.

| Number of histopathology diagnoses | p16^INK4A^ pattern number of specimens | | | p16^INK4A^ intensity /number of specimens | | | HSV pattern number of specimens | | | HSV intensity /number of specimens | | |
| --- | --- | --- | --- | --- | --- | --- | --- | --- | --- | --- | --- | --- |
|  | Lack of p16 expression | Focal  N=36 | Diffuse  N=23 | Int 1 n=23 | Int 2  N=25 | Int 3  N=11 | Lack of p16 expression | Focal  N=13 | Diffuse  N=8 | Int 1  N=19 | Int 2  N=2 | Int 3  N=0 |
| Oral lichen planus without dysplasia n=29 | 11  38.0% | 9  31.0% | 9  31.0% | 3  10.3% | 9  31.0% | 6  20.7% | 28  96.5% | 1  3.5% | 0  0.0% | 1  3.6% | 0  0.0% | 0  0.0% |
| Fibroma  N=81 | 63  77.8% | 15  18.5% | 3  3.7% | 11  13.6% | 6  7.4% | 1  1.2% | 71  87.7% | 7  8.6% | 3  3.7% | 9  11.1% | 1  1.2% | 0  0.0% |
| Mucocele  N=20 | 16  80.0% | 3  15.0% | 1  5.0% | 3  15.0% | 1  5.0% | 0  0.0% | 17  85.0% | 2  10.0% | 1  5.0% | 2  10.0% | 1  5.0% | 0  0.0% |
| Epithelial hypertrophia/ hyperplasia  N=34 | 20  58.8% | 8  23.5% | 6  17.7% | 5  14.7% | 5  14.7% | 4  11.8% | 30  88.2% | 2  5.9% | 2  5.9% | 4  11.8% | 0  0.0% | 0  0.0% |
| Inflammatory infiltration  N=16 | 14  87.5% | 0  0.0% | 2  12.5% | 0  0.0% | 2  12.5% | 0  0.0% | 16  100% | 0  0.0% | 0  0.0% | 0  0.0% | 0  0.0% | 0  0.0% |
| Ulceration  N=6 | 5  83.3% | 1  16.67% | 0  0.0% | 0  0.0% | 1  16.7% | 0  0.0% | 5  83.3% | 1  16.7% | 0  0.0% | 1  16.7% | 0  0.0% | 0  0.0% |
| Squamous papilloma  N=21 | 19  90.4% | 0  0.0% | 2  9.5% | 1  4.8% | 1  4.8% | 0  0.0% | 19  90.5% | 0 | 2  9.5% | 2  9.5% | 0  0.0% | 0  0.0% |
| Others  N=4 | 4  100% | 0  0.0% | 0  0.0% | 0  0.0% | 0  0.0% | 0  0.0% | 4  100.0% | 0  0.0% | 0  0.0% | 0  0.0% | 0  0.0% | 0  0.0% |

χ^2^_14_=37,3 p=0,00067 for p16^INK4A^ pattern distribution

χ^2^_21_=47,9 p=0,00070 for p16^INK4A^ expression intensity
